# Rif1: A Conserved Regulator of DNA Replication and Repair Hijacked by Telomeres in Yeasts

**DOI:** 10.3389/fgene.2016.00045

**Published:** 2016-03-30

**Authors:** Stefano Mattarocci, Lukas Hafner, Aleksandra Lezaja, Maksym Shyian, David Shore

**Affiliations:** Department of Molecular Biology and Institute for Genetics and Genomics in Geneva, University of GenevaGeneva, Switzerland

**Keywords:** Rif1, telomere, DNA replication timing, DNA repair, DNA recombination, telomere capping, Rap1

## Abstract

Rap1-interacting factor 1 (Rif1) was originally identified in the budding yeast *Saccharomyces cerevisiae* as a telomere-binding protein that negatively regulates telomerase-mediated telomere elongation. Although this function is conserved in the distantly related fission yeast *Schizosaccharomyces pombe*, recent studies, both in yeasts and in metazoans, reveal that Rif1 also functions more globally, both in the temporal control of DNA replication and in DNA repair. Rif1 proteins are large and characterized by N-terminal HEAT repeats, predicted to form an elongated alpha-helical structure. In addition, all Rif1 homologs contain two short motifs, abbreviated RVxF/SILK, that are implicated in recruitment of the PP1 (yeast Glc7) phosphatase. In yeasts the RVxF/SILK domains have been shown to play a role in control of DNA replication initiation, at least in part through targeted de-phosphorylation of proteins in the pre-Replication Complex. In human cells Rif1 is recruited to DNA double-strand breaks through an interaction with 53BP1 where it counteracts DNA resection, thus promoting repair by non-homologous end-joining. This function requires the N-terminal HEAT repeat-containing domain. Interestingly, this domain is also implicated in DNA end protection at un-capped telomeres in yeast. We conclude by discussing the deployment of Rif1 at telomeres in yeasts from both an evolutionary perspective and in light of its recently discovered global functions.

## Introduction

Telomeres, the ends of linear eukaryotic chromosomes, pose two fundamental problems for the cell. First, the polarity of DNA synthesis, and its initiation by an RNA primer that must be subsequently replaced by DNA, means that conventional replication mechanisms cannot duplicate the termini of linear molecules (the so-called “end replication problem”; Watson, 1972; Olovnikov, 1973; Lingner et al., 1995). Second, chromosome ends physically resemble accidental DNA double-stranded breaks (DSBs), but must be treated differently by the cell to avoid DNA damage checkpoint activation and the genome instability caused by chromosome end fusions or translocations (the “end protection problem”).

**FIGURE 1:**
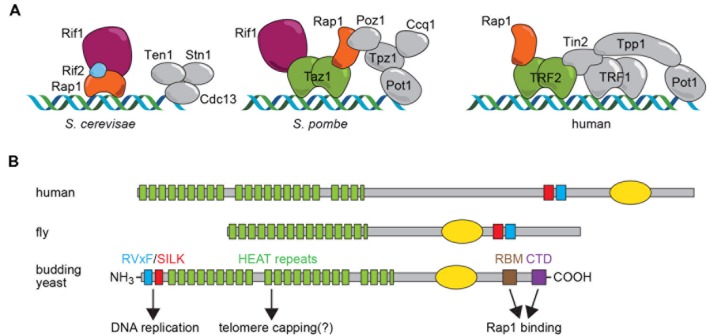
**(A)** Shelterin complexes assembled on telomere-repeat sequences in budding yeast (*Saccharomyces cerevisiae*), fission yeast (*Schizosaccharomyces pombe*) and human cells. Proteins discussed here are highlighted in color. It should be noted that *Schizosaccharomyces pombe* and human also contain a CST complex involved in DNA replication at telomeres and, at least in humans, genome-wide. **(B)** Schematic representation of Rif1 motif structure in human, fly and budding yeast, with functional properties for the *Saccharomyces cerevisiae* protein indicated below. The yellow oval represents a region of homology to the alpha-CTD of bacterial polymerases that in hRif1 has been shown to have DNA-binding activity (Xu et al., 2010).

Organisms with linear chromosomes have thus had to evolve special mechanisms, carried out by a relatively conserved set of proteins, to replicate chromosome ends and to hide them from highly sensitive DNA damage checkpoint and repair systems (de Lange, 2009). In nearly all eukaryotes the end replication problem is solved by the specialized reverse transcriptase enzyme called telomerase, which adds short G-rich repeated sequences [TG_1-3_ and T_2_AC(A)(C)G_2-8_, in *Saccharomyces cerevisiae* (Sc) and *Schizosaccharomyces pombe* (Sp), respectively; T_2_AG_3_ in metazoans] to chromosome 3′ ends, using an intrinsic RNA template. The regulated action of telomerase prevents the continual erosion of chromosome ends with succeeding cell divisions, and allows for the maintenance of a constant average length of telomere repeats at chromosome ends. A conserved complex of six proteins referred to as shelterin protects (or “caps”) chromosome ends in metazoans (de Lange, 2005) thus solving the end protection problem (**Figure 1A**). Although the targets of shelterin throughout evolution appear to be highly conserved (e.g., ATM/ATR checkpoint pathways and the telomerase enzyme), the actual shelterin components themselves are less well conserved in yeasts, particularly in budding yeasts, where only one shelterin component, Rap1, is present (**Figure 1A**).

This Perspective article will focus on the Rif1 (Rap1-interacting factor 1) protein, a telomere-binding protein originally found in the budding yeast *Saccharomyces cerevisiae* (Hardy et al., 1992) and later in the distantly related fission yeast *Schizosaccharomyces pombe* (Kanoh and Ishikawa, 2001). More recently Rif1 has come to be recognized as a highly conserved protein in metazoans (Sreesankar et al., 2012). Surprisingly, though, there is no clear evidence that Rif1 is a telomere binding protein in any multicellular organism. Instead, recent discoveries in mammalian and yeast systems have pointed to two unanticipated and conserved functions of Rif1 that have dramatically altered our view of this protein. These studies reveal that Rif1 acts genome-wide to regulate DNA repair pathway choice and the temporal pattern of DNA replication. In the following sections, the telomeric functions of Rif1 and its more widespread functions will be described with reference to conserved structural domains and motifs in Rif1 (**Figure 1B**). Finally, we will highlight and discuss unresolved questions related to the evolution of Rif1 as a telomeric protein in yeasts.

## Telomeric Functions of Rif1 in Yeasts

ScRif1 was first shown to negatively regulate telomere elongation, based on the observation that telomere repeat tracts in *rif1Δ* cells are on average about twice the length of those in wild type cells (Hardy et al., 1992). A second Rap1-interacting factor, Rif2, has a smaller effect on telomere length and works in a parallel pathway (Wotton and Shore, 1997). The way in which Rif1 and Rif2 assemble on telomeric DNA has recently been elucidated in molecular detail by x-ray crystallography (Shi et al., 2013). Remarkably, both Rif1 and Rif2 employ a short alpha-helical peptide motif, referred as the Rap1-binding module (RBM; for Rif1_RBM_ see **Figure 1B**) to bind to a conserved groove in the C-terminal domain of Rap1 (Rap1_RCT_). Rif1 also contacts Rap1 at a different site on the RCT, though with lower affinity, through a tetramer-forming C-terminal domain (Rif1_CTD_; see **Figure 1B**). Rif2 also contains a second Rap1-interacting domain that makes contact with a third region on the Rap1 C-terminus. This network of Rap1–Rif1–Rif2 interactions thus generates a “molecular Velcro” that promotes the cooperative binding of Rif1/Rif2 to the arrays of DNA-bound Rap1 found uniquely at telomeres (Shi et al., 2013). However, Rap1 binding alone is not sufficient for telomere length regulation by Rif1, since mutations in the conserved RVxF/SILK (involved in PP1 phosphatase binding; see **Figure 1B**) and the HEAT repeat domains cause telomere elongation (our unpublished results). Remarkably, the Rap1-interacting C-terminus of Rif1 is not required for some degree of telomere length regulation (Shi et al., 2013), suggesting that Rif1 may be able to localize to telomeres through a second mechanism, perhaps involving the large, conserved HEAT domain that occupies a significant portion of the Rif1 N-terminus (**Figure 1B**, see below). The targets of Rif1 and Rif2 in telomerase inhibition still remain to be clarified (Bianchi and Shore, 2008; Gao et al., 2010).

Although not essential for capping, recent studies show clearly that ScRif1 plays a role in protecting telomere ends. This was first revealed by its genetic interaction with Cdc13, a telomere-specific single-strand DNA-binding protein that forms part of the RPA-like Cdc13-Stn1-Ten1 (CST) complex essential for capping telomeres in the G2/M phase of the cell cycle (Anbalagan et al., 2011; Xue et al., 2011; see **Figure 1A**). When CST function is compromised, Rif1 becomes essential for telomere protection and survival. Even in cells where CST is perfectly functional, Rif1 is required for checkpoint inhibition at short telomeres (Ribeyre and Shore, 2012), where it works in parallel with Rif2 in the so-called telomeric anti-checkpoint (Michelson et al., 2005). Remarkably, these protective functions of Rif1 also do not require the C-terminal domains necessary for targeting to telomeric DNA through Rap1 interactions (Xue et al., 2011; our unpublished data). These observations point to a possible role of the N-terminal HEAT repeats in localizing Rif1 to its sites of action in chromatin.

In *Saccharomyces cerevisiae*, and indeed in many organisms where it has been examined, chromatin immediately internal to the telomere repeat tracts is transcriptionally silenced, or heterochromatic (Gottschling et al., 1990). This phenomenon, referred to as telomere position effect (TPE), is carried out by a set of SIR (Silent Information Regulator) proteins. SIR proteins are recruited to telomeres though interactions with both Rap1 and the Yku70/80 proteins, and spread along telomere-adjacent chromatin aided by the histone deacetylase activity of the highly conserved Sir2 protein (reviewed in Rusche et al., 2003). Interestingly, Rif1 counteracts the repressive function of SIR proteins at telomeres, at least in part by competing with Sir3, which also contains a RBM, for binding to the Rap1 C-terminus (Kyrion et al., 1993; Buck and Shore, 1995; Wotton and Shore, 1997; Shi et al., 2013). However, in *Candida glabrata*, the only other budding yeast where Rif1’s telomeric silencing function has been examined, TPE is abolished by *rif1Δ*, despite the fact that this mutation has a similar telomere elongation phenotype to that observed in *Saccharomyces cerevisiae* (Castano et al., 2005).

The only other yeast in which Rif1 function has been directly examined, the fission yeast *Schizosaccharomyces pombe*, presents a very different picture. To begin with, SpRif1 is recruited to telomeres through an interaction with Taz1 (also a Myb domain DNA-binding protein, but more similar to human TRF1/TRF2), and not with SpRap1 (**Figure 1A**). Whereas SpRif1 also plays a role in limiting telomere elongation, though via a Rap1-independent pathway, there is no evidence that it prevents telomeres from activating DNA damage response (DDR) pathways (Kanoh and Ishikawa, 2001; Miller et al., 2005). Interestingly, SpRif1 and SpRap1 have opposite effects in *taz1Δ* cells, which are inviable at low temperatures due to chromosome entanglement. Deletion of Sp*Rif1^+^* restores normal growth in *taz1Δ* cells, suggesting that SpRif1 might block telomere recombination (Miller et al., 2005). With respect to TPE, SpRif1 appears to play a positive role at subtelomeric regions (Greenwood and Cooper, 2012).

## Rif1 is a Regulator of DNA Repair

**FIGURE 2:**
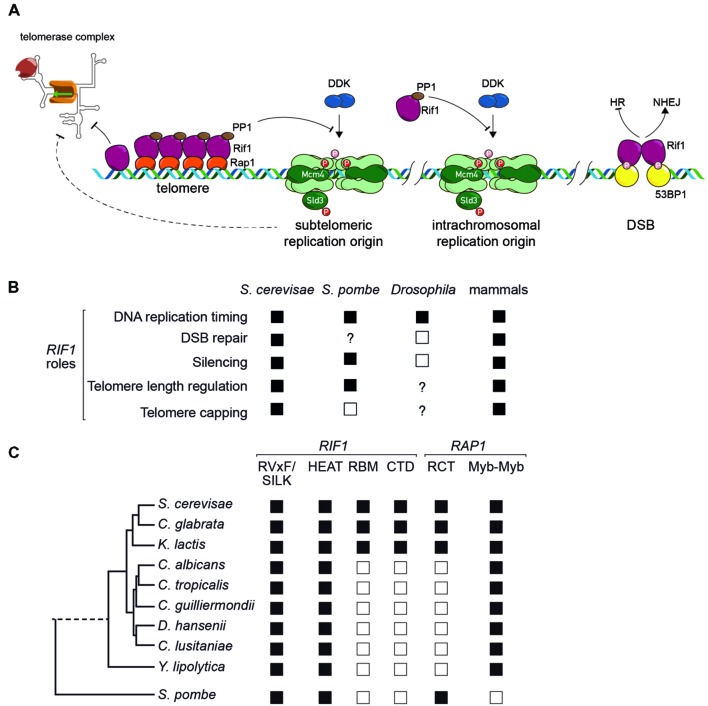
**(A)** Schematic representation of Rif1 function at budding yeast telomeres (left), replication origins in yeasts (center) and double-strand breaks in mammalian cells (right). See text for details. **(B)** Conservation of Rif1 functions across species. Filled squares indicate that the function is present, according to at least one report; open squares indicate evidence for absence of function in at least one report; “?” indicates that the function has not been tested for in that organism. Note that for mammals, a Rif1 role in silencing, telomere length regulation and telomere capping has been examined only in mouse ES cells (Dan et al., 2014). **(C)** Conservation of Rif1 and Rap1 domains in yeasts. Filled square indicates presence and open square absence of indicated domain or motif.

Building upon the early observations that human Rif1 (hRif1) localizes to damaged telomeres (Silverman et al., 2004; Xu and Blackburn, 2004) and also contributes to survival under DNA replication stress (Buonomo et al., 2009), a flurry of more recent reports have provided new molecular insights into the role of both human and mouse Rif1 in the DDR (Chapman et al., 2013; Di Virgilio et al., 2013; Escribano-Diaz et al., 2013; Zimmermann et al., 2013). Together, these studies showed that Rif1 is recruited to DNA double-strand breaks (DSBs) through an N-terminal phosphorylated domain of 53BP1, with which it cooperates to block DSB resection (**Figure 2A**). This action of Rif1 promotes break repair by non-homologous end-joining (NHEJ) in the G1 phase of the cell cycle and is opposed by the action of BRCA1 in S phase, which permits a switch to a homologous recombination (HR) mode of DNA repair. Given that HR is less error-prone than NHEJ, this conversion allows cells to profit from the availability of an intact sister chromatid during S phase.

Contrary to initial reports (Xue et al., 2011), it now appears that budding yeast Rif1 also localizes to DSBs (Martina et al., 2014; our unpublished results), strongly implying a role for Rif1 in some aspect of the DDR. Although yeast cells deleted for *RIF1* do not display any obvious increase in sensitivity to agents that damage DNA, the *rif1Δ* mutation displays “synthetic” phenotypes in combination with some mutations affecting replication or repair pathways, such as the MRX (Mre11-Rad50-Xrs2) complex, which is involved in both HR and NHEJ-mediated repair (Costanzo et al., 2010; Guenole et al., 2013; Martina et al., 2014). However, the precise role of Rif1 in the DDR in yeast cells is still not clear. Martina et al. (2014) have recently presented evidence that Rif1 promotes resection in yeast, thus, in principle, favoring HR over NHEJ.

## Rif1 Controls the Temporal Pattern of DNA Replication Initiation Through the PP1 Phosphatase

One striking phenotype to emerge recently in studies of *RIF1* deletions in budding and fission yeasts, as well as knock-down experiments in mouse and human cells, is a global effect on the temporal pattern of chromosomal DNA replication. In all eukaryotes studied to date, replication in most cell types initiates at characteristic sites (origins) whose “firing” can occur either early during S phase, or at middle or late periods. This temporal pattern of replication initiation is highly controlled, but the underlying mechanisms are still poorly understood. The finding, that *rif1Δ* cells in both budding (Lian et al., 2011; Peace et al., 2014) and fission (Hayano et al., 2012) yeasts display major alterations in replication timing, was thus of considerable importance. Similar results were reported in studies of mouse and human cells in culture that were depleted for Rif1 (Cornacchia et al., 2012; Yamazaki et al., 2012). In *Schizosaccharomyces pombe* and mammalian cells the effects of Rif1 on replication timing were widespread, whereas in budding yeast initial studies suggested that they might be more restricted to telomere-proximal regions, where most late-firing origins are found.

Several lines of evidence provided clues to the mechanism by which Rif1 influences replication timing. The first of these, mentioned above, was the finding by Sreesankar et al. (2012) of the conserved SILK/RVxF motifs in Rif1, suggesting that the protein might serve as a PP1 phosphatase co-factor or recruitment scaffold. A second key finding made in both fission and budding yeast, was that deletion of *RIF1* permits the growth of mutants with reduced Cdc7 (SpHsk1) protein kinase activity (Hayano et al., 2012; Dave et al., 2014; Hiraga et al., 2014; Mattarocci et al., 2014). Cdc7/Hsk1 kinase is the catalytic subunit of the Dbf4-dependent kinase (DDK) required for activation of the pre-Replication Complex (pre-RC). This genetic interaction suggests that Rif1 acts as a negative regulator of a process promoted by the DDK (**Figure 2A**). As predicted by this model, phosphorylation of two DDK targets in the pre-RC, Mcm4, part of the replicative helicase, and Sld3, a conserved adaptor protein involved in assembly of an active DNA polymerase on the pre-RC, is increased in point mutants affecting the Rif1 SILK/RVxF motifs (Dave et al., 2014; Hiraga et al., 2014; Mattarocci et al., 2014). Interestingly, suppression of *CDC7* mutation in budding yeast also requires the Rif1 HEAT motif region (Hiraga et al., 2014).

Given the presence of SILK/RVxF motifs in all Rif1 homologs, from yeast to human, it is tempting to speculate that the Rif1–PP1 interaction is ubiquitous. Indeed, this conclusion is supported by biochemical findings in human cells (Trinkle-Mulcahy et al., 2006). A strong prediction from the studies in both fission and budding yeast, but yet to be tested, is that SILK/RVxF mutations in mammalian Rif1 homologs will be defective in the PP1 interaction and display aberrant patterns of DNA replication.

One important mechanistic question that is still not fully understood is how Rif1 action is targeted so as to affect some but not all origins. In budding yeast this is partly resolved, since as pointed out above Rif1 localizes to telomeres through a network of interactions with Rap1, and firing of subtelomeric origins is strongly inhibited by Rif1 (see **Figure 2A**). Nevertheless, normally dormant chromosome-internal origins are activated in *rif1Δ* cells and there is so far no indication of how (or even if) Rif1 is targeted to these sites. In *Schizosaccharomyces pombe*, one very recent study provides evidence that Rif1 is recruited through an interaction with G-quadruplex DNA structures (Kanoh et al., 2015). An even more recent study in mouse embryonic stem cells (ESCs) indicates that Rif1 acts at the level of nuclear architecture to constrain late-replicating chromosomal domains to interact with each other exclusively during the period in G1 when replication timing is established (Foti et al., 2015).

## A Common Thread in Rif1 Function Throughout Evolution?

Recent studies thus now point to control of DNA replication initiation and DNA repair as highly conserved functions of eukaryotic Rif1 homologs (**Figures 2A,B**). The likely conservation of the Rif1–PP1 interaction throughout evolution, as well as the replication initiation targets identified in budding yeast (Mcm4 and Sld3), suggests that this Rif1 function may be the most conserved in mechanistic detail. The conservation of Rif1’s function in the DDR is presently less clear. Here the role of mammalian Rif1 is better defined, with its recruitment to sites of damage requiring an interaction with 53BP1. In budding yeast the 53BP1 homolog, Rad9, counteracts the function of Rif1 (Martina et al., 2014), perhaps explaining why Rif1 in yeast appears to promote, rather than block 5′ end resection, at least in G1 cells. We find it interesting that data from both yeast and human cells are beginning to point to a role for the highly conserved HEAT repeat domain of Rif1 in localizing Rif1 to sites of damage (Xue et al., 2011; Escribano-Diaz et al., 2013). Although a C-terminal conserved domain with DNA-binding properties has been implicated in efficient hRif1 recruitment at stalled replication forks (Xu et al., 2010), the function of this domain in the DDR is still controversial (Escribano-Diaz et al., 2013). Furthermore, the possible role of the Rif1–PP1 interaction in the DDR has yet to be explored. Finally, the more general question of a possible relationship between the replication timing and DNA damage/repair functions of Rif1 has yet to be addressed. In this regard it is worth noting that replication provides sister chromatids that can facilitate homologous repair

## Appropriation of Rif1 at Yeast Telomeres: How and Why?

As pointed out above, and illustrated in **Figure 1A**, Rif1 appears to be localized to native (capped) telomeres only in yeasts. Yet again, though, the evolutionary scenario leading to this situation is uncertain, due to the different mechanisms for Rif1 telomere recruitment employed by fission and budding yeasts. In the budding yeast *Saccharomyces cerevisiae*, Rif1 localizes to telomeres through a network of interactions with ScRap1, as detailed above. However, the SpRif1 does not require SpRap1 for telomere binding, but instead localizes to telomeres through an interaction with Taz1, the duplex DNA telomere-binding protein in this organism. Thus, the most conserved partner of Rif1 in yeast shelterin complexes, Rap1, is not universally used for its recruitment. This curious fact may be explained by the observation that Rap1 probably emerged as a direct duplex DNA telomere-binding protein only in the *Saccharomycotina* yeasts where its Myb-like DNA-binding domain underwent duplication (**Figure 2C**). The budding yeasts still retain a Taz1/TRF2-like protein, called Tbf1, which itself retains telomere-capping functions (Ribaud et al., 2012). One plausible evolutionary scenario is that Taz1/Tbf1 recruited Rif1 to telomeres in the last common ancestor of fission and budding yeasts, with Rap1 acquiring this function as it replaced Tbf1 as the telomere-binding protein in budding yeasts. However, this scenario leaves open the question of how Rif1 is recruited by Rap1 in the large number of *Saccharomycotina* clades (including the well studied human pathogen *Candida albicans*) where Rap1 has no recognizable RCT domain (**Figure 2C**). Significantly, the Rif1 homologs in these organisms lack recognizable RBM and CTD domains (**Figure 2C**), implying that, if Rap1 does indeed recruit Rif1 to telomeres in these organisms (yet to be demonstrated experimentally), it does so through a different set of interactions.

It is interesting to consider what selective advantage telomeric Rif1 localization might afford to yeasts. One possibility is that modulation of replication timing at sub-telomeric regions by Rif1 provides a mechanism to regulate telomerase action as a function of telomere length, at least in part because early replication, which occurs at short telomeres, permits increased elongation in a given cell cycle (Bianchi and Shore, 2007) (**Figure 2A**). This may be particularly advantageous in yeasts where telomere repeat tracts are more than an order of magnitude shorter in length than in mammals and often have an irregular repeat sequence, both of which may limit t-loop formation. In addition, Rif1’s still poorly understood end-capping function (Xue et al., 2011; Ribeyre and Shore, 2012; Martina et al., 2014) might also contribute to telomerase regulation (**Figure 2A**). It is also worth noting that the late-replicating sub-telomeric regions in *Saccharomyces cerevisiae* are at least partly heterochromatic and serve as a niche for gene families that play an important role in environmental adaptation (Brown et al., 2010). Their late replication causes a higher rate of mutagenesis (Lang and Murray, 2011), which has been speculated to confer a selective advantage in fluctuating environmental conditions.

As a closing word of caution, we note that the unique presence of Rif1 at native telomeres in yeasts might be more apparent than real. It is possible that Rif1 is present at capped telomeres in metazoans, but in low amounts that have so far escaped detection, perhaps because it acts transiently during telomere replication and/or reassembly of the telomere cap, or in cell types that have not been carefully studied. In this regard it is worth noting that Rif1 is highly expressed in mouse ESCs and a recent report suggests that it is telomere-localized in these cells, where it plays a role in sub-telomeric heterochromatin formation (Dan et al., 2014). Interestingly, it appears that Rif1 represses a gene, *Zscan4*, a gene whose product promotes HR at telomere repeats. It seems clear that we are only beginning to understand the various functions of Rif1, much less their underlying mechanisms and evolutionary origins. The recent interest that Rif1 has attracted in both the DNA replication and DNA repair fields suggests that the coming years will bring new and important discoveries about this remarkably multifunctional protein.

## Author Contributions

SM, LH, and AL made equal contributions to this work. All authors listed have made substantial and direct intellectual contributions to this work and have approved it for publication.

## Conflict of Interest Statement

The authors declare that the research was conducted in the absence of any commercial or financial relationships that could be construed as a potential conflict of interest.
